# Case report: actinomycosis of the abdominal wall

**DOI:** 10.1093/jscr/rjab171

**Published:** 2021-05-05

**Authors:** Mario Tarzi, Alma Douedari, Rama Aldakhil, Aghyad Kudra Danial, Ahmad Al-Haj

**Affiliations:** Faculty of Medicine, University of Aleppo, Aleppo, Syria; Faculty of Medicine, University of Aleppo, Aleppo, Syria; Faculty of Medicine, University of Aleppo, Aleppo, Syria; Department of Surgery, University of Aleppo, Aleppo, Syria; Department of Surgery, University of Aleppo, Aleppo, Syria

## Abstract

Primary abdominal wall actinomycosis is rare, but even rarer when it comes to intestinal infiltration; it is usually misdiagnosed as a neoplasm in computed tomography till proved otherwise with pathological examination.

We report a 59-year-old diabetic male presented with a year-old abdominal wall mass, diagnosed by pathology after surgical excisional biopsy, and treated with penicillin for 6 months. We recommend consideration of actinomycosis in cases of abdominal wall mass, especially in immunocompromised patients, as a differential diagnosis of neoplastic lesions.

## INTRODUCTION

Actinomycosis is a rare, slowly progressive and granulomatous disease caused by *Actinomyces* species, which colonizes the oral cavity, gastrointestinal tract and vagina [1]. Extension to adjacent structures in immunosuppressive patients is common.

It presents with weight loss, malaise, change in bowel habits, abdominal pain and palpable abdominal mass, which might lead altogether to a misdiagnosis of neoplasia [2]. Its mimicking features of tumors make it challenging to come up with a preoperative diagnosis based on radiological tests. Thus, microbiological culture is a gold standard diagnostic test. However, positive results are quite rare, leading to a greater challenge in coming up with an accurate diagnosis [3]. Histopathological examination of biopsy remains applicable in clinical practice whenever it is needed to differentiate between neoplasm and actinomycosis, also in cases of negative results obtained by microbiological culture.

Pathological features include classical sulfur granule arrangement with extensive fibrosis and granulomatous tissue formation [4].

We present a case of a 59-year-old male patient with a record of 15 years of diabetes and hypertension, who developed primary actinomycosis in the abdominal wall with intestinal infiltration.

## CASE PRESENTATION

A 59-year-old male patient presented with moderate, intermittent pain at the periumbilical region for 1 month. It was accompanied with a gradually increasing mass over a year time. His bowel habits were altered, and abdominal pain was relieved after defecation. There were no other symptoms concerning nausea, vomiting or weight loss. The patient denied any history of abdominal trauma, surgery or injection at that region. On the other hand, his medical history was eventful. He had type 2 diabetes and hypertension for 15 years. He was treated with oral antidiabetics and angiotensin converting enzyme inhibitors (ACEIs). The patient neither smokes nor drinks alcohol.

Upon his first visit, his pulse and blood pressure were normal. His physical examination revealed 10 cm distention in the right upper quadrant of the abdomen that crossed the midline. Its size did not change upon increased intra-abdominal pressure. The patient was afebrile with no signs of local inflammation.

The mass was firm, non-pulsatile, painless, with no clear borders and could not be detached from the innards, still it moved with breathing movements. The overlying skin was moveable as well.

The radiologist performed ‘Focused Assessment Sonography Test’ to rule out any existence of free fluid in the abdomen corresponding with trauma or any systemic disease. The test showed neither presence of free fluid nor detected any abdominal mass, which was irrelevant to the fact of the visual bulge.

Computed tomography (CT) scan depicted a solid irregular density 15 × 11.5 × 6 cm sized, occupying bilaterally musculus rectus in the umbilical area. The lesion was projecting through anterior abdominal wall lipoid tissue and posterior mesenteric lipoid tissue. The mass had a heterogeneous characteristic; 2 cm of the bowel was infiltrated within the mass. It also showed no enlargement of lymph nodes or other organs (Fig. 2).

## INTERVENTION

**Figure 1:**
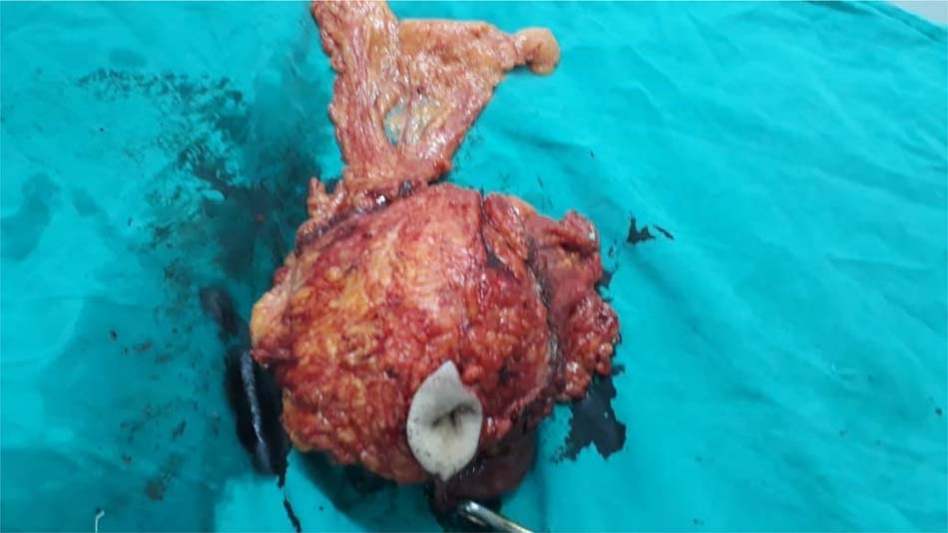
The mass after surgical removal. It consisted of the umbilicus, the tumor itself, a part of the omentum and intestines.

With respect to previous results, we suspected neoplastic origin of the mass and decision was made for excisional biopsy with regards to safety borders (Fig. 1).

**Figure 2:**
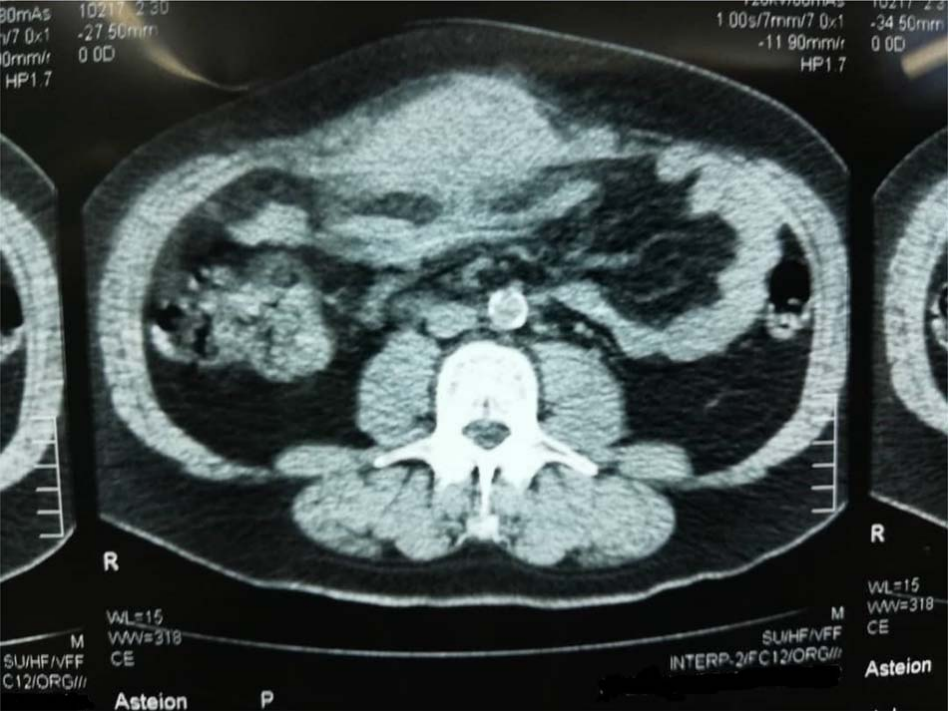
CT scan of the tumor as it shows infiltration to adjacent structures and attachment to the anterior abdominal wall.

**Figure 3:**
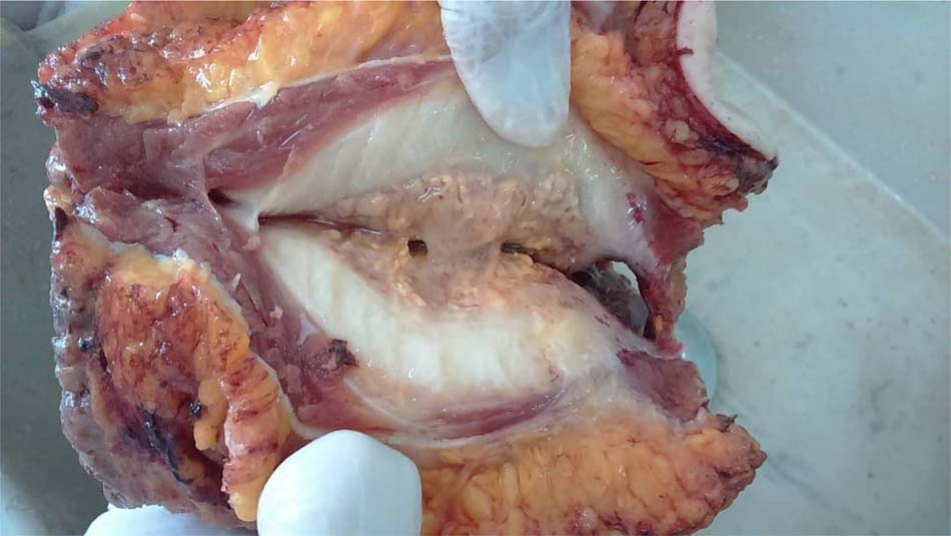
Gross anatomical view of the mass after dissection; note the jelly substance in the middle.

**Figure 4:**
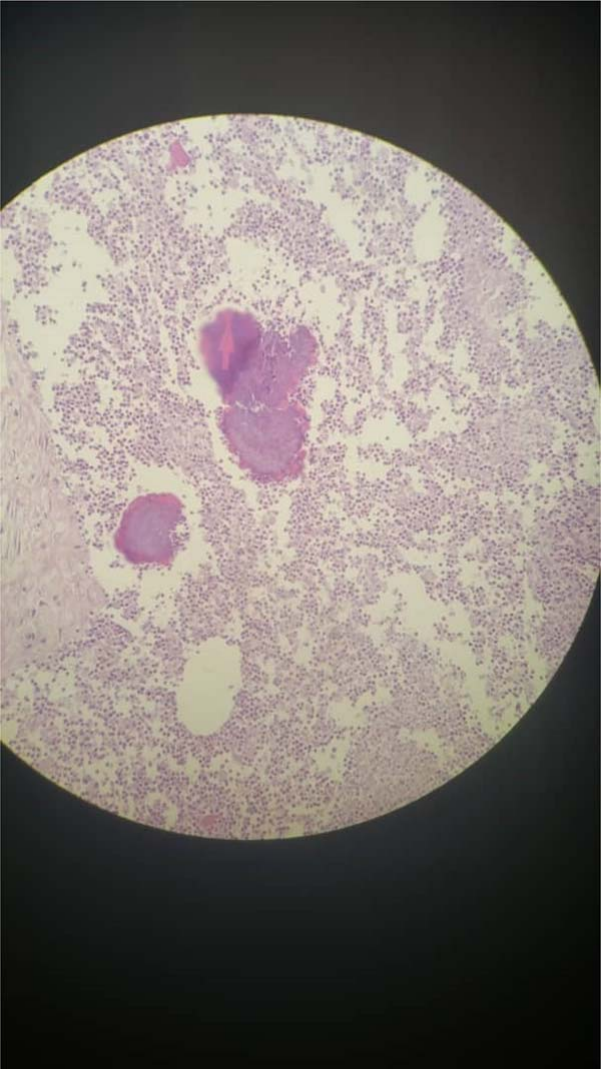
Microscopic evaluation of the tumor specimen.

Microscopic examination showed benign stratified squamous epithelium, surrounded by skin with wide area of fat necrosis and abscess formation, composed of massively acute inflammatory infiltrate of neutrophils, eosinophils, foamy cells, histocytes and plasma cells with many ‘sulfur’ granules, which contain irregular round clusters of bacteria rimmed by eosinophilic, club-like projections of proteinaceous material (Splendore–Hoeppli material) (Fig. 4). Colon and small intestine sections revealed benign mucosal layer, submucosal layer and muscular propria with wide areas of fibrosis and granulation tissue (Fig. 3). Presence of five reactive lymph nodes; no evidence of malignant cells.

All above was suggestive of actinomycosis.

Treatment consisted of parenteral crystalline penicillin 24 million units/day for 1 month, followed by oral penicillin V for 6 months.

## DISCUSSION

*Actinomyces* species are members of normal flora in immunocompetent individuals. However, they might act as pathogens whenever an impaired state of immunity is detected. So the majority of cases are seen in immunocompromised statuses also in intestinal necrosis, abdominal operations and implementation of intra-abdominal devices such as intrauterine contraceptive device [2, 5, 6]. We present a case of a male who has been treated for diabetes mellitus for 15 years.

The infection involves cervicofacial (50%), abdominopelvic (20%) and thoracic (15%) regions [5, 7]. However, the implication of the abdominal wall, as mentioned in our case, is rare. Abdominal wall actinomycosis usually occurs in the rectus abdominis muscle, with involvement of underlying layers such as peritoneum and lipoid tissue. Extension to abdominal organs is even seldom. In our case, the primary lesion has initiated in the abdominal wall and has later infiltrated in the peritoneal cavity. Gross section of the biopsy revealed involvement of the omentum, small intestine and colon (Fig. 3).

Abdominal mass accompanied with unspecific digestive symptoms and CT findings oriented the decision to make an excisional biopsy. Pathology results denied any malignancy while confirming the inflammatory nature of it.

Actinomycosis is an infectious disease treated with antibiotics, mainly penicillin G [8, 9]. Treatment is usually initiated with surgical debridement followed by antibiotic course. The reported regimen is penicillin G 10–24 million units for 2–6 weeks, followed by oral penicillin V 25–30 mg/kg for every 6 h for additional 6–12 months [3, 10, 11]. Surgery remains controversial in the common practice, yet required in specific cases including symptoms affecting patient’s daily activities, recurrence and when there is suspicion of malignancy [12].

Due to the suspicion of the neoplastic origin, decision was made to remove the lesion. The histopathological study revealed actinomycotic infection. Antibiotic treatment was prescribed as mentioned by the previous regimen.
